# Dietary Fibers: Shaping Textural and Functional Properties of Processed Meats and Plant-Based Meat Alternatives

**DOI:** 10.3390/foods13121952

**Published:** 2024-06-20

**Authors:** Aleksandra Marczak, Ana C. Mendes

**Affiliations:** Research Group for Food Production Engineering, Technical University of Denmark (DTU)-Food, Henrik Dams Allé B202, 2800 Kgs., 2800 Lyngby, Denmark

**Keywords:** dietary fibers, meat, plant meat alternatives, plant ingredients, meat analogs, texture, food, food innovation

## Abstract

The search for alternative sources of plant-based ingredients to improve the textural and sensory properties of plant-based meat alternatives (PMAs) is a growing trend, with the potential to enhance the sustainability of global food systems. While much focus has been placed on plant-based proteins, it is known today that dietary fibers (DFs) can also play a key role in the textural and other physicochemical properties of traditional processed meat products and PMAs. This review examined the latest scientific literature regarding the advantages of using DF in food. It showcases the latest applications of DF in processed meats, PMAs, and the effects of DF on the functional properties of food products, thereby aiming to increase DF applications to create improved, healthier, and more sustainable meat and PMA foods. The predominant effects of DF on PMAs and processed meats notably include enhanced gel strength, emulsion stability, improved water-holding capacity, and the formation of a uniform, porous microstructure. DF also commonly enhances textural properties like hardness, chewiness, springiness, and cohesiveness. While the impact of DF on processed meats mirrors that of PMAs, selecting the right DF source for specific applications requires considering factors such as chemical structure, solubility, size, concentration, processing conditions, and interactions with other components to achieve the desired outcomes.

## 1. Introduction

There is a high demand for plant-based meat alternatives (PMAs), with a predicted expansion of the plant-based food market from 11.3 billion USD (2023) to 35.9 billion USD (2033) [1]. Plant-based meat alternatives (PMAs) hold the potential to support the transition from animal meat consumption to meat-less foods [2], and can fit different social dietary habits. The challenges in PMAs are mainly related to the tentative replication of meat sensory properties, including texture. Most of the current issues to produce suitable textured PMAs are linked to:

(i) Variability in the plant ingredients and their functionalities: Different ingredients grant different textural functionalities to the PMAs. During the formulation stage, plant proteins are combined with other components (such as fats, water, thickening agents, colorants, flavorings, and binders) or nutrients (such as vitamins or minerals [3].
(ii) Lack of fibrous structure: The fibrous structure of animal muscle plays a crucial role in the texture of meat. PMAs are often composed of pea protein, soy protein, wheat protein, or a combination of these [2]. Pea and wheat proteins do not create meat-like fibrous structures and thus require specific technologies to develop a meat-like fibrous texture, such as extrusion cooking [4], shear cell processing, 3D printing, and spinning technologies [3,5].
(iii) Meat quality: textural properties such as chewiness and juiciness. Juiciness is a not well-understood property, and thus is difficult to be replicated by PMAs. Achieving the right level of chewiness in plant-based meat is another significant challenge [2].

Although plant proteins have been receiving great focus to produce PMAs, today, it is known that dietary fibers (DFs) also exhibit interesting opportunities for modeling and improving the texture, and other functional properties of PMAs. The meat industry has been including DF in their hybrid and processed meat products to create healthier, tastier, and more sustainable processed meat products [6,7], and therefore, this knowledge could be applied in PMAs.

DF exists naturally in plant-based foods such as cereals, vegetables, fruits, and nuts, in which the DF amount and composition vary from food to food [8]. Higher amounts of DF (>50% fibers on a dry base) can be found in a larger number of food industrial by-products such as in the pomace of tomatoes, hazelnut skin, mango, apple, grape, orange by-products [9], and sugar beet pulp, which are often wasted. The amount of wasted food accounts for 3.3 billion tons of CO_2_ emissions [10], whereas cereals, vegetables, and fruits account for most of the global food waste and food loss. Those are rich in many functional ingredients, including DF sources such as cellulose, lignin, and pectin. Upcycling that waste could create new economic benefits and improve the sustainability of the food production systems by recovering fiber from waste (circular economy) [11].

Increasing the consumption of dietary fibers could benefit public health [12,13], positively impact the sustainability of the food systems, and improve the functionality and quality properties of conventionally processed meat products and PMAs.

Despite existing studies on the effects of dietary fibers (DFs) in meats or hybrid meats, there is still a gap in the literature regarding articles that can disclose the link between the effects of DF on the texture of plant-based meat analogs (PMAs) and processed meat. Specifically, there is a lack of studies examining the effects of DF on both types of products and exemplifying the application of DF in the product formulation.

Therefore, this review aims to elucidate the latest developments (spanning from 2019 to 2024) in enhancing the functional attributes of hybrid processed meats and plant-based meat alternatives (PMAs) through the integration of dietary fibers. Additionally, it seeks to provide readers with insights drawn from the extensive existing knowledge on the effects of dietary fibers (DFs) on meat, enabling them to anticipate functionalities in plant-based meat analogs. This review also uniquely attempted to provide an overview of DF sources applied commercially to PMAs, which may aid food formulators in designing new products. With a specific emphasis on texture enhancement, the objective is to promote the increased utilization of dietary fibers in crafting healthier and more sustainable food options, thereby enhancing consumer acceptance.

### 1.1. Dietary Fiber: Classification and Health Benefits

**Figure 1:**
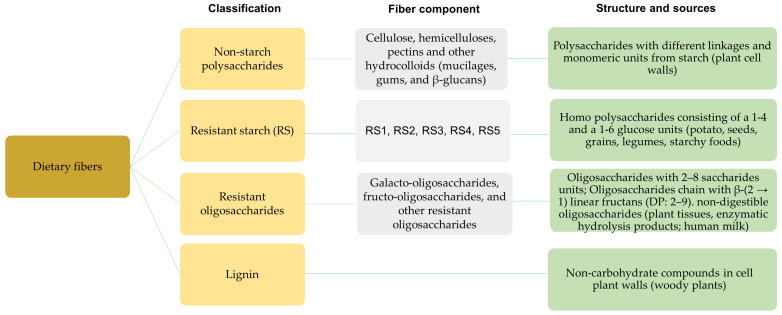
Classification of dietary fibers, structures, and sources [14].

Among the different classifications of DFs, which might entail DF’s solubility, fermentation [8,14], and digestion properties, EFSA classifies DF into four subgroups of non-digestible carbohydrates and lignin [15,16], as depicted in Figure 1. This classification includes all non-digestible carbohydrates due to their important nutritional effect related to small intestinal digestibility. According to the classification, undigestible oligosaccharides must contain a minimum chain length of three monomeric units. Furthermore, non-starch polysaccharides were grouped. However, it should be noted that non-starch polysaccharides vary in molecular sizes, structures, monomeric compositions, and physiological effects [14].

DFs have many known health benefits: fiber fermentation gives rise to several short-chain fatty acids and reinforces intestinal immunity by promoting the growth of acidophilic lactic acid bacteria over pathogenic species [15]. Its high water-holding capacity improves mineral absorption and glycemic response and reduces plasma cholesterol [16]. The intake of DF could also contribute to weight management [16,17]. Furthermore, the deficiency of DF is usually associated with an increased risk of chronic diseases, such as type 2 diabetes, cardiovascular diseases, and colorectal cancer [15].

The recommended daily intake of DF in Europe is 25 g [14], and most adults in Europe do not meet that target. Therefore, the enrichment of food products with DF would benefit public health [12]. Conventional meat products do not contain significant amounts of dietary fiber. On the contrary, meat substitutes may be a source of dietary fiber, as some of the PMAs may contribute to around 16.68% of the daily recommended intake for DF [17].

### 1.2. Dietary Fibers and Their Functional Properties in Foods

DFs vary greatly in their properties; therefore, their choice of product formulation will affect the final texture of the product [14]. DFs are known to affect hydration; increase viscosity; form gels; and change the shelf life of the product and sensory attributes (flavor, texture, and color) in food products [13]. Those functional properties are dependent on DF solubility, porosity, particle size, shape, and arrangement [15]. For example, DFs derived from fruits and vegetables have a relatively high proportion of soluble DF, which gives them superior gelling ability, hydration properties, oil-holding capacity (OHC) [18], and water-holding capacity (WHC). WHC is linked to the hydrophilic nature of DF. Soluble DF entraps water in the three-dimensional network of polysaccharide molecules linked to water molecules through hydrogen bonds [19]. OHC is attributed to their large surface area and porosity that binds DFs to lipids [15]. Examples of DF sources commonly used in foods include individual components such as pectin; glucomannan; carrageenan; alginate; inulin; resistant starch; fructooligosaccharides; polydextrose; carboxymethyl cellulose; hemicellulose; lignin; or composites such as bran, pomace, peel, and vegetable by-products [20]. At the same time, a wide array of DF sources not so commonly used exists with promising functionalities in PMAs, such as psyllium husk [21], oat fiber [22], Belgian endive fiber [12], sunflower cake [23], and citrus fiber. In many products, DF has been shown to increase the quality parameters of meats and PMAs, such as the cooking yield, emulsion stability [19,21], juiciness, and shrinkage [20], which benefits the economic gain as well [24]. Ultimately, as mentioned, adding DF to meat and PMA products can enhance their nutritional profile. 

To evaluate the effect of DF on the texture and other functional properties of meats and plant-based meats, various instrumental analyses have been performed. The methods are divided into mechanical (e.g., tensile or compression and puncture test, etc.); spectroscopy (e.g., Fourier-Transform Infrared Spectroscopy, Near-InfraRed, Middle InfraRed, Raman, Nuclear Magnetic Resonance, and light reflectance); and imaging-based (e.g., visual, Confocal Laser Scanning, Scanning Electron, Transmission Electron, and Atomic Force Microscopies). The examples of methods and their applications were reviewed previously by Schreuders et al. [24]. The most frequently used mechanical method to measure textural properties is the Texture Profile Analysis (TPA). TPA allows the assessment of textural features such as hardness, adhesiveness, chewiness, cohesiveness, resilience, and springiness. Those textural properties are related to the microstructure; the type (e.g., proteins, lipids, and DF); the amount of the ingredients [20]; and the properties of the foods, such as the water-holding capacity, moisture, and cooking loss. These textural properties may also be evaluated from the perspective of the eating experience, which requires sensory analysis. For instance, sensory analysis revealed that various commercial PMAs were perceived as less chewy, cohesive, fibrous, juicy, and fatty, with a weaker meaty flavor, compared to authentic beef burgers [2]. These perceptions were consistent with TPA measurements of hardness, chewiness, and cohesiveness. Thus, the instrumental textural properties are, in some cases, of great relevance to predicting the optimal sensory characteristics of PMAs [2].

## 2. Effect of Dietary Fibers on the Texture of Plant-Based Meat Alternatives

PMAs often have lower hardness, chewiness, and cohesiveness than their meat counterparts [20]. However, upon the addition of DF to PMAs, the most common effects include increased hardness, chewiness, improved moisture retention or WHC, baking yield, stronger gels, or more stable emulsions. For instance, the addition of Belgian endive fiber to plant-based soy protein burgers increased the hardness and also improved the baking yield and moisture retention [12]. In a comprehensive review that included 13 commercial chicken PMA and 14 burger PMAs, a higher content of various DF sources applied by producers in chicken PMAs was positively correlated with TPA hardness and chewiness values, while it was negatively correlated with cohesiveness. A higher proportion of fiber in PMA chickens contributed to a higher moisture content but also a greater cooking loss. In PMA burgers, the inclusion of a higher proportion of fibers was correlated to lower expressible moisture, which could be due to different water-holding properties of fibers [2]. As for the most frequently applied DFs, non-starch polysaccharides such as hydrocolloids are often reported to improve the structural and textural properties of PMAs. For instance, the study by Zhang et al. successfully replicated the textural attributes and microstructure of real scallops using a blend of pectin from citrus fiber, yellow pea flour, and transglutaminase. The inclusion of 0.5% pectin facilitated the production of an uncooked scallop analog with high TPA hardness and chewiness. After grilling, the TPA parameters (hardness, cohesion, springiness, and chewiness) of the scallop analog were not significantly different from those of real scallops. Additionally, Scanning Electron Microscopy revealed that the microstructure of the analog with added pectin resembled that of a real scallop, displaying a honeycomb structure that was porous and rough [25].

In another example of the application of hydrocolloids to PMAs, the combination of xanthan gum with kappa-carrageenan or guar gum enhanced the textural properties of a gluten-free extruded soy protein-based PMA [26]. The addition of salts to the hydrocolloids formulation also contributed to changes in the texture of the PMA. For instance, high acyl gellan gum (HG), low acyl gellan gum (LG), high methoxyl pectin (HP), low methoxyl pectin (LP), and xanthan were applied with CaCl_2_ and NaCl at different concentrations [27]. Overall, the addition of hydrocolloids resulted in large (in the case of HG) or intermediate fibers (in the case of the other hydrocolloids).

In addition, DFs recently found applications to produce plant-based fat analogs that can be used in PMAs. Wen et al. developed a fat analog of 3D-printed potato starch and inulin. These fat analogs simulated animal fat behavior throughout the cooking process. The improved melting behavior was hypothesized to be a result of the gelatinization properties of starch [28]. The texture of 3D-printed PMA was also shown to benefit from the addition of insoluble dietary fiber from okara (residue of soybean processing) [29]. Soy protein isolate-based PMA produced with high-moisture extrusion also benefited from the addition of insoluble DFs at 10–20 wt%, as it facilitated the formation of a fibrous structure and mechanical anisotropy.

The effects of DFs in foods are dependent on their physicochemical properties and their interactions with the other ingredients in the food matrix. Although the addition of DF can often lead to predictable changes in some of the textural profiles of a PMA, there are also some unexpected findings. For instance, and contrary to most of the findings reported so far, the incorporation of oat fiber concentrate in an extruded pea protein-based PMA reduced the PMA structural strength (hardness and chewiness) [22].

Processing technology and the formulation of PMAs also have a great influence on the final textural properties. For instance, various hydrocolloids were applied to pea protein–wheat gluten PMA produced via shear cell technology. The anisotropic index, micro- and macrostructure, tensile strength, rheological behavior, and WHC among samples with different hydrocolloids were compared, and it was suggested that low acyl gellan gum and xanthan gum had the greatest potential to improve the textural properties of PMAs [30]. On the contrary, Dinani et al. showed that low acyl gellan gum compromised structural improvements, produced short and thin filaments, and decreased tensile strength in soy protein PMAs obtained via high-temperature shear cell technology. However, insoluble soy fiber applied to the same PMAs increased air retention and thus structural integrity and produced elongated fibers, which resulted in improved elasticity and tensile strength [29].

Furthermore, DF powder’s size affects the PMA properties, as the size may influence the oil- and water-binding ability, which, in turn, affects the texture and mouthfeel of the product [19].

An overview of the recent studies of DF applied to PMAs is shown in Table 1.

### Industrial Relevance of Dietary Fibers in Plant-Based Meat Alternatives

The demand for healthier food products, the increased awareness of the DF health benefits, and the functional properties of DF in foods, particularly in PMAs, have been simulating the DF market growth from USD 12,343.6 million in 2024 to USD 33,098.0 million in 2034 [36]. Today, many commercial PMAs use DFs (Table 2).

The amount of dietary fiber (DF) in a product is important to consumers from a nutritional perspective. For instance, in the EU, products can be labeled as a “source of fiber” if they contain at least 3 g of fiber per 100 g of product, and “high in fiber” if they contain at least 6 g of fiber per 100 g of product [62]. From the examples listed in Table 2, it is evident that most commercially available PMA products worldwide contain less than 6 g of dietary fiber per 100 g of PMA. However, it is feasible to achieve higher amounts of DFs (>6 g/100 g in PMA), as demonstrated by some commercial products listed in Table 2. The most widely applied DF is methylcellulose (MC). According to producers, MC is applied due to its thickening and stabilizing functionalities. Recent research shows that other fibers, e.g., enzymatically treated pea, citrus, or apple DF, can maintain the same level of taste, texture, juiciness, and WHC as MC [63]; therefore, MC is likely applied mostly due to its low price. In 2024, the MC price ranged between 2.40 and 3.20 USD/kg [64]. Another popularly applied DF to PMAs is carrageenan, due to its emulsifying and thickening properties. Products of higher DF contents often apply a mixture of hydrocolloids, e.g., methylcellulose, and other fibers, e.g., pea fiber, wheat fiber, oat fiber, or inulin, likely due to the different textural effects that can be obtained by mixing these DF types.

## 3. Effect of Dietary Fibers on the Texture of Hybrid and Processed Meat

DF has been included in meat formulations as an attempt to decrease global meat consumption and improve the nutritional value of meat products without impairing the sensory experience. Examples of meat products containing DFs include burgers, sausages, cutlets, and lean meat products where DF successfully replaced portions of animal fat [65]. DF in processed meat products has been used as binders and fillers to replace the animal fat components of the products and increase acceptability through the improvement of the WHC, shear force, and nutritional and sensory properties [7]. Furthermore, DFs have been demonstrated to play a key role in the textural properties of meat products, mainly due to:

1. Moisture retention [66], which can be seen particularly for DFs with high WHC or DFs with gel-forming capabilities. DF with a high WHC tends to decrease the cooking loss of meat and increase the emulsion stability [18]. DFs with gel-forming properties can also retain water in their 3D gel network and improve the overall mouthfeel of processed meats. DF in meat products also improves the cooking yield (due to a high WHC) [6].
2. Binding and structural support, depending on the type of DF source, can hold meat pieces together in processed products like burgers, sausages, and meatballs. Improving the structure of processed meat can enhance textural properties like cohesiveness [67], reduce cooking losses [67,68], and improve the overall stability [69] and yield of processed meats over time and their mouthfeel [6].
3. Fat replacement: fats contribute to the texture and mouthfeel of meat, and fibers can mimic some of these properties. Some DFs have also been applied as fat mimetic ingredients in reduced-fat meat products due to their favorable particles, droplet sizes, and ability to influence the rheological properties, as well as to stabilize emulsions [13].
4. Stabilization of fats and proteins [66], which increases the product shelf-life.

In animal meat, juiciness and tenderness are among the most important textural properties and are also associated with a meat’s quality [20]. The WHC, hardness, and cooking loss are often related to the perceived juiciness of meats [2,70,71]. These properties are strongly related to the structure of the skeletal muscle, which is composed of myofibrillar proteins (myosin and actin) [20]. The DF inulin was shown to modify the secondary structure of porcine myosin [72], enhancing its heat-induced gelation. This suggested that textural changes in meat can be explored by examining the structural and functional alterations in myofibrillar proteins. Furthermore, the gelation properties of myofibrillar protein were positively influenced by psyllium [73], citrus fiber [74], or coconut kernel fiber [75]. Another DF that showed a positive influence on myofibrillar protein gelation is modified cellulose, a derivative of microcrystalline cellulose. It is also characterized by high WHC and gel properties with an appearance and mouthfeel comparable to animal fat [76]. Furthermore, DF is often applied as a fat replacement in hybrid meat products. For instance, oat hull fiber was applied to beef burgers to replace fat, partially or fully (T2 burger). The sensory panel was recruited and consisted of sixty regular meat-consuming participants. Overall, 59.32% of the panelists preferred the T2 burger over the regular beef burger. The T2 burger was significantly lower in all TPA parameters; hence, the authors suggest that the softer texture of the T2 burger resulted in its preference over the control [77].

Besides purified DF, whole plant flours rich in DF (e.g., white bean flour) were successfully applied in hybrid meat lean pork burgers and exhibited similar textural properties to pork and a more stable gel matrix [65]. The inclusion of lesser amounts of DF coming from flours rich in DF can result in a decreased hardness and chewiness of certain meat products [65] and increase their tenderness and juiciness [77].

Table 3 gives an overview of the recent studies about DF in animal processed meat products and their effects on the texture and other meat functional properties.

## 4. Modulatory Effects of Dietary Fibers: Opportunities and Current Challenges

**Figure 2:**
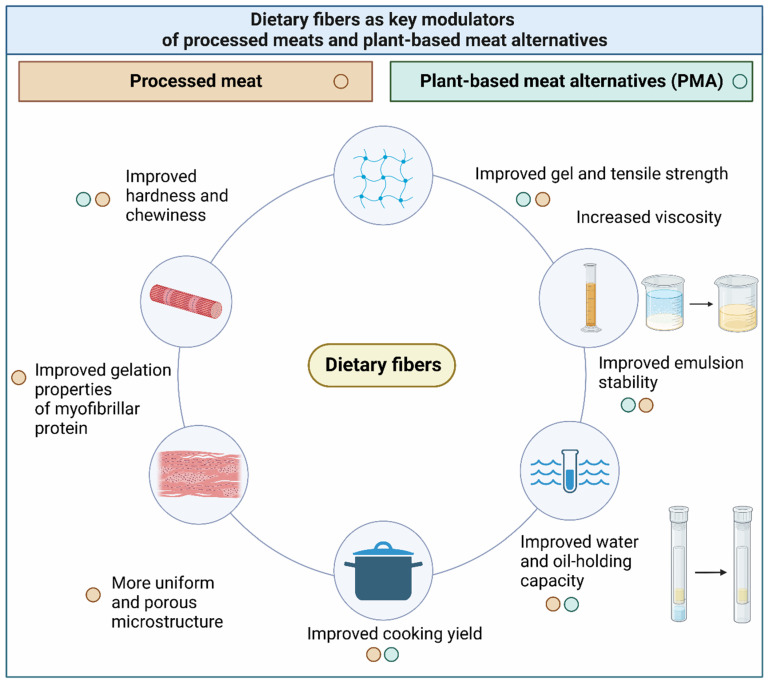
Schematic representation of the effect of dietary fibers on the functional properties of conventional processed meats and plant-based meat alternatives (PMAs).

DF has been proven to exert different effects on the functional properties of processed meats and PMAs, which are summarized in Figure 2. However, it is to note, as discussed above, that, due to the wide variety of DFs (Figure 1), the effects described in Figure 2 are dependent on the source, the type of DF, DF concentration, and potential interactions with the other ingredients of the food/meat matrix.

The desirable structure and overall functional textural properties exerted from the DF source have also been associated with DF molecular interactions with proteins. Interactions with DF polysaccharides are known to play a crucial role in modifying the functional properties of food proteins, such as solubility, surface activity, conformational stability, gel-forming ability, emulsifying properties, and foaming properties. Furthermore, when processing technologies that use temperature (e.g., extrusion cooking) are applied, protein denaturation exposes previously hidden reactive sites in the protein molecular chains, increasing the structural flexibility, which facilitates configurational adjustments, and enhancing the protein-polysaccharide interactions. Furthermore, polysaccharides can function as crosslinkers, altering protein conformation and binding to protein side groups through the Maillard reaction, and other types of interactions polysaccharide-protein, thus forming a protein network structure [82]. 

Several studies have shown that dietary fibers, even in a small amount, could largely affect the functional properties of gluten-containing properties of products [82]. This is mostly attributed to the interactions between gluten and DFs, which involve changes in protein secondary and tertiary structures, disulfide bridges, and hydrogen bonding. Depending on the solubility of the DF, different types of interactions can be anticipated. Most of the soluble DFs interact noncovalently with gluten through hydrogen bonding and hydrophobic interactions, though it remains unclear whether electrostatic interactions are predominant for anionic soluble fibers. For insoluble fibers, interactions are partly driven by the degree of swelling and hydration. Additionally, physical mechanisms such as water competition and steric hindrance play significant roles comparable to chemical factors, like the molecular weight of soluble fibers and the water-binding capacity of insoluble fibers. Taking into account that many PMAs and processed meats also use starches containing gluten or gluten ingredients, understanding how gluten proteins interact with DFs to control the structure and related physicochemical properties will help PMAs with enhanced functionality.

In another study, it was shown that incorporating legume flours and pea fiber can modify the functionality of pea protein-based formulations, leading to plant-based meat products with diverse textures after low moisture extrusion [31]. The water absorption capacity showed a significant increase compared to the pea protein control when pea fiber was added to a pea protein/legume flour blend, rising by 8–16%. Furthermore, the addition of fiber led to a more cellular and porous microstructure, resulting in a bulk density 55% to 58% lower than the control. These differences in bulk density and water capacity, influenced by the porosity and layering effects, affected the instrumental texture characteristics of the ground-hydrated product. Specifically, the hardness increased due to higher layering caused by the starch addition but decreased due to the porosity induced by fiber incorporation. 

The potential of DF (insoluble soybean fiber) on the soybean protein isolate emulsion properties and stability was also investigated. Increasing the insoluble soybean fiber content improved the emulsions’ resilience to pH and salt fluctuations while enhancing the storage stability [83]. Herein, the fiber promoted the stability of soy protein isolate emulsions due to electrostatic interactions, the formation of a gel-like network structure, and due to the DF emulsifier and thickener properties. This facilitated the adsorption of the protein onto the oil droplet surface and enhanced the interfacial protective coating. Overall, insoluble soybean fiber showed promise for use in emulsion systems with low-protein concentrations, reducing the need for additional protein while enriching the nutritional value with insoluble dietary fiber.

For instance, peanut protein powder was blended with various DF exogenous polysaccharides (e.g., carrageenan and sodium alginate) and wheat starch to study the development of anisotropic structures [84]. The results showed that extrudates based on 0.1% sodium alginate had the highest fibrous index, although they also exhibited increased hardness and chewiness. The selected exogenous polysaccharides, particularly in the presence of wheat starch, can promote protein molecule aggregation by breaking intramolecular disulfide bonds, enhancing hydrophobic interactions, and increasing the apparent viscosity to stabilize the new protein conformation. These polysaccharides promote the unfolding of the α-helix, gradually converting it into β-turns and random coil structures. When pea protein powder is mixed with 0.1% carrageenan, the protein secondary structures in the extrudate follow the order: β-sheet > α-helix > β-turn > random coil. However, with 0.1% sodium alginate or 2% starch, the order is β-sheet > β-turn > α-helix > random coil. Herein, in this study, it was demonstrated that the addition of DF to a protein can enhance the structure and, consequently, the texture of PMAs and hybrid processed meats. However, the extent of the effect is dependent on the DF chemical structure. 

Although the application of DFs in PMAs and processed meats has been increasing, the application of DFs in foods can be challenging for food manufacturers [36]. Those challenges are mainly associated with (i) taste: many DF sources have an earthy and bitter aroma that is not pleasant for consumers’ experience; (ii) amount: although it is recommended to increase the DF intake, still, increasing the amounts of certain types of DFs, if it exceeds a certain amount in foods, it can compromise negatively textural properties of PMAs and the taste of the PMAs [33]; (iii) cost: DFs of high quality/purity can be costly; (iv) DF solubility: DFs can be both water-soluble and -insoluble, which can create challenges to disperse/integrate DFs in certain PMA food matrices and create homogeneous products with higher consumer acceptance; and (v) E-numbers assigned to many dietary fibers discourage consumers from purchases [4].

## 5. Conclusions

This review paper summarizes the modulatory role of various DFs on the texture and other functional properties of processed meats and PMA products. The most frequently reported textural changes to meat products after the application of DFs include increased gel strength, emulsion stability, improved WHC, a more uniform and porous microstructure, and an enhanced cooking yield. DF is also often reported to improve textural properties such as hardness, chewiness, springiness, and cohesiveness. However, the effect varies depending on the meat product, DF type, and concentration; therefore, improvement of these properties relies on experimentation. In low-fat systems, DF was often reported to have a positive impact on sensory meat juiciness.

The challenges of the PMA alternative producers to recreate meat-like textures have encouraged researchers to explore the advantages of using DFs in these products. Examples of DFs commercially applied in PMAs include various hydrocolloids, such as methylcellulose, carrageenan, and various gums. The application of other dietary fibers is less widespread, even though research on DFs such as oat, psyllium, citrus fiber, and Belgian endive fibers has also been shown to improve the textural properties of PMAs. Those DFs come from various husks, hulls of seeds and grains, gums, and various fiber-rich flours of beans and legumes. Inulin is widely described by the literature as an excellent meat fat replacer. 

The effects of DFs on PMAs’ texture depend on the DF type, processing technology, and ingredients used in the formulation. Textural properties of PMAs can be strengthened or loosened upon interactions with other food matrix components and are often quantified concerning a control food product, which dictates the magnitude of the effect of DF on the texture. Overall, DF tends to increase the hardness and chewiness in PMAs, while its effect on juiciness is still a textural property not well understood, requiring more research in this field. Furthermore, it has been quite evident that DF often increases the WHC, oil-holding capacity, gel, tensile strength, and viscosity of PMAs, often without noticeable color changes. 

Overall, one can anticipate that the effects of DFs on the functional properties of processed meats are similar to the ones observed for PMAs. However, the selection of a DF for a given food should consider its source, chemical structure, solubility, size, concentration, processing condition, and interactions with other food components to achieve the desired textural and functional outcomes.

## Figures and Tables

**Table 1:** Applications and effects of dietary fibers (DFs) in plant-based meat alternative (PMA) products.

DF	PMA Product Type	DF Amount (% *w*/*w*)	DF Effect on the PMA Texture	DF Effect on Other Functional Properties	Reference
Belgian endive (inulin, pectin, and insoluble DF fraction)	Plant-based burgers (soy-based)	0, 5, 10	Increased hardness, decreased cohesiveness, and had no significant effect on springiness and chewiness. Shear force was increased significantly by 5% DF. Sensory chewiness increased sequentially with a concentration of DF, while cohesiveness decreased.	Replacing soy protein with DF improved baking yield, moisture retention, and diameter reduction. Sequential inclusion of DF increased sensory intensity of overall taste and odor. Burgers with the inclusion of DF appeared darker than the control.	[12]
Oat fiber concentrate (β-glucan and insoluble DF fraction)	Extruded fibrous meat alternative based on pea protein	25, 50, 75	DF reduced the mechanical strength (hardness, chewiness, springiness, gumminess) of meat alternatives and decreased void fraction. 30–50% of DF could be incorporated without impairing meat-like fibrous structures and viscosity properties of DF.	ND	[22]
Pea fiber (pectin and insoluble DF fraction)	Extruded meat alternative based on pea protein isolate (PPI) and legume (chickpea, pea flour)	5, 10, 15	The addition of fiber resulted in a more porous, cellular microstructure, and 55–58% lower bulk density compared to control. Due to porosity caused by DF, the hardness of the alternative decreased. DF had no significant effect on springiness.	Pea fiber added to meat alternatives increased WAC by 8–16%. Pea fiber reduced the mutual dampening effect of starch from flour and protein, as peak viscosity increased while peak temperature remained constant.	[31]
Sweet potato stems fiber (insoluble dietary fibers)	Vegetable patty analogues	0, 10, 20, 30, 40, 50% of pea protein weight	The fiber bundles integrated between protein networks allowed the soft texture of PMA. TPA parameters decreased with the DF concentration. DF relaxed the rigid control structure.	Emulsion stability was improved by the inclusion of DF in the vegetable patty analog. Lightness and redness (a*) values decreased over fiber concentration, while yellowness value (+) increased.	[32]
Psyllium husk (PH); Psyllium husk powder (PW), Psyllium seeds (PP)	Plant-based sausages of texturized pea protein and chickpea flour	3, 4, 5, 6	Psyllium increased all TPA parameters, except adhesiveness. The highest cutting strength was obtained with PW. The presence of psyllium increased the consistency of the continuous phase of sausage emulsion.	Significantly improved (decreased) water release. The acceptability of PW in concentrations 4% and 6% was the highest among samples including the control. Color change of PW sausages was the least of the samples. PW treatment significantly increased the content of carbohydrates, and ash and decreased fat content. PP caused significant (*p* < 0.05) color changes and low scores in acceptability by the sensory panel.	[33]
Citrus pectin	Seafood scallop alternative based on pea protein	0, 0.5, 1	Pectin did not have a significant effect on springiness and cohesion. On the other hand, pectin at a concentration of 0.5% significantly (*p* < 0.05) increased hardness and chewiness. Textural properties assessed with the TPA of alternatives were similar to those of the conventional scallops.	WHC decreased over pectin concentration. Color values were not significantly different between the scallop analog and conventional scallop.	[25]
Guar gum (G), κ -carrageenan (C), xanthan gum (X), hydroxypropyl starch, cross-linked tapioca starch	Soy protein-based, gluten-free, extruded meat analog	1, 2, 3, 4, 5, 6, 7	Increased viscosity and degree of plasticity. G and C enhanced hardness, springiness, cohesiveness, and gumminess.	ND	[26]
Xanthan (X), iota-carrageenan, sodium alginate, guar gum (GG), carboxymethyl cellulose (CMC), low acyl gellan gum (GZ), low methylated pectin, locust bean gum(LBG)	Meat analogs of pea isolate and wheat gluten obtained by shear cell technology	1, 2, 3	All fibers substantially increased the anisotropy index of PMA. However, only X, CMC, and GG fibers produced the most strongly flow-oriented and fibrous. GZ resulted in significantly (*p* < 0.001) higher tensile stress. X and GZ were suggested to be promising hydrocolloids to improve the textural properties of PMA.	Only X substantially increased the WHC of PMA. The inclusion of X, GG, and LBG at all concentrations increased the browning index of PMA, especially at 3%, significantly (*p* ≤ 0.001) compared to the control.	[30]
Iota- carrageenan	Pea protein, wheat gluten blended PMA produced by shear cell technology	2	DF increased WHC, tensile stress, and number of air bubbles	Increased browning index. The quality of PMA was increased without the need to hydrate iota-carrageenan beforehand.	[34]
Insoluble dietary fiber (IDF) from okara	PMA based on soy protein isolate and wheat gluten	0–10	Improved texture (increased WHC, elongation at break, hardness, gumminess, chewiness cohesiveness, and tensile strength), improved PMA structure	The incorporation of 10% IDF resulted in optimal printability and printing accuracy. IDF promoted the formation of hydrophobic and disulfide bonds.	[35]

ND—no data, TPA—Texture Profile Analysis, WHC—water-holding capacity, and WAC—water absorption capacity.

**Table 2:** Examples of commercial PMA products with dietary fibers (DFs).

Product Type	Product Name	Company	DF Used/ Nutritional Content (g) per 100 g of PMA	Reason to Use DF	Country of Origin	Link to the Product
Sausage	NATURLI’ Sausages	NATURLI’ FOODS A/S	Pea fiber, methylcellulose/ 4.5 g	Stabilizer: methylcellulose	Denmark	[37]
Sausage	IMPOSSIBLE^®^ SAUSAGE	IMPOSSIBLE FOODS Inc.	Methylcellulose/1.8 g	ND	US	[38]
Sausage	BEYOND SAUSAGE^®^	BEYOND MEAT Inc.	Inulin, methylcellulose, psyllium fiber/ 1 g per sausage	ND	US	[39]
Sausage	Future Sausage	Fazenda Futuro^®^	methylcellulose, carrageenan/ 1.5 g	Stabilizer: methylcellulose, thickener: carrageenan	Brazil	[40]
Sausage	ACCRO’s plant-based sausage	ACCRO	Pea fiber, sodium alginate, guar gum, konjac gum, methylcellulose/ 5.4 g	Stabilizers: sodium alginate, guar gum, konjac gum, methylcellulose	France	[41]
Sausage	Plan*t^®^ spicy chorizo	Sustainable Foods Limited	Methylcellulose, pea fiber/<9.1 g	ND	New Zealand	[42]
Meatball	BEYOND MEATBALLS^®^	BEYOND MEAT Inc.	Methylcellulose/1.2 g per meatball	ND	US	[43]
Meatball	Damhert Vegan No Meat Boulet	Damhert Nutrition N.V.	Methylcellulose, carrageenan, wheat fiber/ 5.7 g	Thickeners: methylcellulose, carrageenan	Belgium	[44]
Meatball	Meeat Food Tech Oy	MUU Balls	Cellulose, methylcellulose/ 4.1 g	Stabilizers	Finland, Estonia	[45]
Meatball	HappyVore	Plant-based and gourmet meatballs	Plantain fiber, maltodextrin, methylcellulose/4.1 g	Stabilizer: methylcellulose	France	[46]
Meatball	The Fry Family Food Co.^®^	No-meat balls	Methylcellulose, bamboo fiber/ 2.6 g	Thickener: methylcellulose	South Africa	[47]
Meatball	Hoya Next Meat CO., LTD.	Plant-based meatball	Methylcellulose/ <8.5 g	ND	Taiwan	[48]
Burger	BEYOND^®^ Burger	BEYOND Meat	Methylcellulose/ 0.9 g	ND	US	[49]
Burger	IMPOSSIBLE^®^ BURGER	IMPOSSIBLE^®^ FOODS	Methylcellulose/ 4.4 g	ND	US	[50]
Burger	The Original Veggie Burger	Big Mountain Foods^®^	Pea fiber/ 13 g	Clan label	Canada	[51]
Burger	Unconventional Burger	Unconventional^®^	Citrus fiber, methylcellulose/ 3.8 g	Stabilizer: methylcellulose	Italy	[52]
Ground Beef	Plant mince	GreenRebel-Foods^®^ Pte Ltd.	Carrageenan, dietary fiber/ 13.8 g	Emulsifier: carrageenan	Indonesia	[53]
Ground Beef	Plant-based minced meat	Yumeat^®^ Denis Asia Pacific PTE LTD	Guar gum/ <6.0 g	ND	Singapore	[54]
Ground Beef	Plant-based minced meat	Let’s Plant Meat	Methylcellulose, carrageenan/ 4 g	ND	Thailand	[55]
Ground Beef	Mince Meat	Switch Foods International LLC	Pea fiber, methylcellulose/ 4 g	Thickener: methylcellulose	United Arab Emirates	[56]
Chicken	Deliciou plant-based chicken	Deliciou LLC	Methylcellulose, inulin/9.2 g	ND	Australia	[57]
Chicken	Vegan Chicken Nature	Endori	Corn fiber, dextrose, potato fiber, psyllium husk, guar gum/ca. 4.6 g	Thickener: guar gum	Germany	[58]
Chicken	Chick’n Fillet Chunks	The Plant-it Food Co.	Methylcellulose, xanthan gum, carrageenan, guar gum/2.7 g	Thickener: methylcellulose	Ireland	[59]
Chicken	High Fibre Chick’n	PHUTURE^®^ FOODS SDN BHD	Oat fiber, apple fiber, wheat fiber, methylcellulose/ 13 g	Unlocking sumptuous taste and creating mouthfeel	Malaysia	[60]
Chicken	Impecable Chicken Breast	The Vegetarian Butcher^®^ by Unilever^®^	Methylcellulose, konjac gum, processed Eucheuma seaweed (carrageenan), citrus fiber, dextrose/ 4.8 g	Thickeners: methylcellulose, konjac gum, processed Eucheuma seaweed (carrageenan),	The Netherlands	[61]

ND—no data.

**Table 3:** Application and effects of dietary fibers in processed meat products.

DF	Meat Product	DF Amount (% *w*/*w*)	DF Effect on the Meat Texture	DF Effect on Other Meat Functional Properties	Reference
A fiber-rich fraction from quinoa wet milling (pectin and insoluble fiber fraction)	Bologna-type sausage	3	Improvement of the emulsion stability. Non-significant differences in the textural properties (TPA measurements), except cohesiveness, decreased (*p* < 0.05), comparatively to control sausages (without DF).	Lightness (L*), redness (a+), yellowness (b+), and saturation index (C*) significantly decreased compared to the control, whereas hue (h*) value significantly increased. DF decreased lipid oxidation and water activity and increased residual nitrate levels, which could lower the need to add additional nitrates.	[78]
Inulin	Porcine myosin gel	0, 1, 2, 3, 4, 5	Gel strength, storage modulus (G′) and loss modulus (G″) of myosin gradually increased with inulin concentration.	Significant (*p* < 0.05) improvement of microstructure (more compact, uniform, porous: inulin 2%. Inulin 1–3% significantly improved WHC (*p* < 0.05). Inulin improved heat-induced gelation of myosin.	[72]
Psyllium	Myofibrillar protein	0.1, 0.5, 1, 2, 3	Low concentrations (0.1–2%) of psyllium increased textural properties (gel strength, adhesiveness, hardness, chewiness)	Increase in WHC (Psyllium 0.1–2%).	[73]
Rice husk DF (insoluble fibers), soybean hull DF (pectin and insoluble fiber fraction), and inulin	Myofibrillar protein gel	1.40, 1.42, 3.24	DF improved significantly (*p* < 0.05) gel strength, WHC, and storage modulus (G′) of MP gel.	DF improved significantly WHC (*p* < 0.05).	[79]
Oat hull (β-glucan and insoluble DF fraction)	Low-fat beef burgers	Replacement of 50% of fat, 100% of fat	100% replacement of fat resulted in a softer texture due to a significant (*p* < 0.05) decrease in hardness, cohesiveness, gumminess, chewiness, and improved juiciness (assessed by sensory analysis).	Full replacement of beef fat significantly increased cooking yield. The color of burgers after cooking was comparable, only the burger with 100% fat replacement was significantly lower than the control in yellowness (b+).	[77]
Potato dietary fiber (pectin and insoluble fiber fraction)	Chicken patties	0, 1, 2, 3, 4	DF facilitated a more homogenous and dense protein-meat network structure. As DF concentration increased the diameter, volume, and weight increased, and the thickness of patties decreased. Over DF concentration, hardness, chewiness, and gumminess significantly increased (*p* < 0.05).	Significantly improved water and fat binding (*p* < 0.05). DF did not affect the thermal denaturation of proteins. DF in concentration 3% did not impair the sensory quality of the patties.	[69]
Rice bran (insoluble fibers)	Hybrid pumpkin-carrot-chicken patties	0, 2, 4, 6, 8	Hardness and gumminess significantly increased with an increase in fiber concentration (*p* < 0.05). Cohesiveness was significantly higher than the control at DF concentrations of 4%, and 6%. Adhesiveness gradually decreased with the concentration of DF.	Cooking loss, moisture, and expressible moisture significantly decreased over DF concentration (*p* < 0.05). Up to 2% of DF could be added without significantly impairing sensory quality. DF significantly lowered pH at concentrations of 6% and 8%. DF significantly lowered the lightness, redness, and yellowness of the patties, but no clear relation between the concentration of DF was observed.	[67]
Resistant Starch (RS), Polydextrose (POD), Fructooligosaccharides (FOS) Galactooligosaccharides (GOS)	Chicken nuggets	5, 10, 15	Firmness was significantly (*p* < 0.05) increased by RS fiber, while decreased by other fibers. Cohesiveness was not affected by other fibers, only significantly decreased by POD. POD, FOS, and GOS significantly decreased Chewiness. POD, and GOS, significantly decreased resilience. Nuggets with 15% RS or GOS had comparable texture, to control (sensory).	The lightness of chicken nuggets was significantly (*p* < 0.05) affected by the level of all types of DF. The total color difference was affected significantly only by RS fiber. pH was not significantly affected by any of the fibers. Sensory evaluation showed that nuggets with 15% RS or GOS had comparable color, and taste to the control. Cooking yield was decreased significantly by POD fiber.	[68]
Kiwi fruit pomace (insoluble fiber fraction)	Low-fat pork meatballs	0, 0.5, 1, 3, 5, 7	Hardness gumminess, and chewiness were significantly (*p* < 0.05) decreased by low concentrations of DF (0–3%) and increased by higher concentrations. Springiness was not significantly affected, except for a significant decrease at a concentration of 7%. The cohesiveness ratio was not significantly affected.	The cooking yield increased gradually over the concentration of DF. Acceptability of appearance decreased over DF concentration (sensory evaluation). A concentration of 3% of DF was found to be most acceptable among the samples according to the sensory panel. The juiciness of meatballs was increased significantly by 7% of DF. The lightness of meatballs decreased over DF concentration. Redness and yellowness on the other hand increased.	[80]
Barley flour (β-glucan, insoluble fiber fraction), Maize flour, Pea hull powder (pectin and insoluble fiber fractions), Wheat bran (β-glucan, fructan, insoluble fiber fractions)	Buffalo meat fillets	Barley flour (12%), maize flour (10%), pea hull powder (8%), wheat bran (8%)	No significant differences between control and fillets with DF were observed in textural properties (hardness, adhesiveness, springiness, cohesiveness, gumminess, chewiness).	All DF slightly increased cooking yield and moisture content. pH of meat products was slightly increased by DF.	[81]

ND—no data, TPA—Texture Profile Analysis, and WHC—water-holding capacity.

## Data Availability

No new data were created or analyzed in this study. Data sharing is not applicable to this article.
